# Whither the Book Retailing Industry in China: A Historical Reflection

**DOI:** 10.1007/s12109-018-9569-0

**Published:** 2018-02-08

**Authors:** Zheng Liu

**Affiliations:** Department of Sociology, University of Cambridge, Free School Lane, Cambridge, CB2 3RQ UK

**Keywords:** Bookselling, Xinhua bookstore, Private bookseller, Online retailer, Revival, China

## Abstract

This article reviews the evolution of the book retailing industry in the mainland area of the People’s Republic of China since the late 1970s. It charts the history and development of the three principal types of booksellers in the general interest book market—the state-owned Xinhua bookstores, private booksellers, and online retailers. In doing this, the article hopes to consider some of the industry’s latest developments and its future by reflecting on them in a historical light.

## Introduction

Over the last three to four decades, the field of book retailing in China has changed in an unrecognizable manner. It has transformed from a field monopolized by the state-owned Xinhua bookstores between the 1950s and 1970s into one in which private booksellers accounted for over half of the general interest book market during the 1990s, and one in which online retailers, owned by large Chinese or foreign corporations, sell more books to consumers than do the high street retailers combined. How did all this happen? Why is it that when faced with competition from online retailers, Xinhua could largely maintain its trade position, but privately run bricks-and-mortar bookstores wilted and declined precipitously? Is the fate of the private bricks-and-mortar bookseller doomed? And how exactly did online retailers become popular in China and what impact have they had on the Chinese retail book market? I address these questions in this article.^1^

**Fig. 1:**
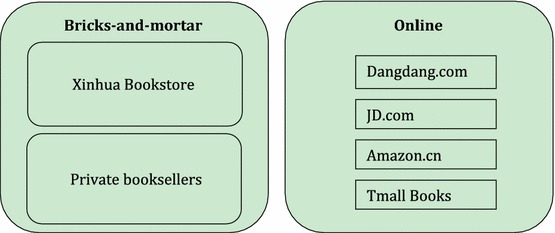
Book retailers in China

But first, let me begin by defining a few concepts that are key to our understanding of the book retailing business in China. Generally speaking, retail booksellers in China today are either bricks-and-mortar booksellers (*shiti shudian*) or online retailers (*wangluo shudian*). Most online booksellers are private companies but customarily people do not talk about ‘private’ or ‘state-owned’ online booksellers, as ‘online booksellers’ are *vis*-*à*-*vis* physical booksellers. The term ‘private bookseller’ (*minying shudian*) therefore refers specifically to the privately owned bricks-and-mortar booksellers. Parallel to private booksellers are the Xinhua bookstores, China’s state-owned bookseller (*guoyou shudian*). Some Xinhua companies have an online presence as well (winxuan.com, owned by Xinhua Winshare Publishing and Media Co., Ltd, for instance), but the majority of Xinhua’s business is completed through the numerous physical Xinhua shops. Figure 1 illustrates this categorization. In terms of sales, Xinhua, private booksellers, and online retailers constitute the three principal book retail channels in China. Other channels such as publishers’ own retail outlets, post offices, and supermarkets have negligible market share compared with the three main channels. For this reason, in this article I focus my attention on examining these three major types of book retailers, and demonstrate how they have developed and changed over the past 30–40 years.

### The Xinhua Bookstore

The ‘Xinhua bookstore’ is the state-owned bookseller in China. Today, there are about 9000 Xinhua bookstores across China, which can generate over ¥70 billion (Chinese yuan) sales each year. Xinhua bookstores are organized on a provincial basis—that is, all Xinhua bookstores in a province are owned by the provincial Xinhua bookstore company. Most provincial Xinhua bookstore companies operate like a chain, especially in terms of centralized stocking. (Centralized stocking is usually organized by *sheng dian*, or ‘provincial head store’, which is responsible for ordering and distributing books to all lower-level Xinhua bookstores in its province except for those in the province’s capital city, which are coordinated and supplied by *shi dian*, or ‘municipal head store’, which operates independently of *sheng dian*). But member Xinhua bookstores are subsidiary companies rather than chain stores of the provincial Xinhua Bookstore company, and many Xinhua bookstores manage their own lower-level branches. A provincial Xinhua bookstore company is often a member of the large publishing group in its province. For instance, Zhejiang Xinhua Bookstore Corporation, which controls all Xinhua bookstores within Zhejiang Province, is a subsidiary company of Zhejiang Publishing United Group.

In the past, all Xinhua bookstores were *shiye danwei*, or public sector organizations, which means that they were fully financed by the government. For most of the three decades from the mid-1950s, Xinhua operated as a centralized national organization, led by the Xinhua General Store in Beijing. In 1987, the centralized Xinhua Bookstore system was dissolved. The Xinhua General Store was turned into a large distributor and retailer managed by the central government (and then merged into China Publishing Group in the early 2000s), and all local Xinhua bookstores were taken over by their provincial governments. The decentralization of Xinhua was accompanied by other structural and organizational reform of the bookseller, one of which was to turn the individual Xinhua bookstores from fully subsidized public ‘cultural institutions’ into self-financing business entities. By the early 2000s, most Xinhua bookstores had completed the reform and become for-profit enterprises. Yet, despite now being mutually independent companies, all Xinhua bookstores have retained the name ‘Xinhua’, which indicates their unchanged nature as state-owned enterprises.

**Fig. 2:**
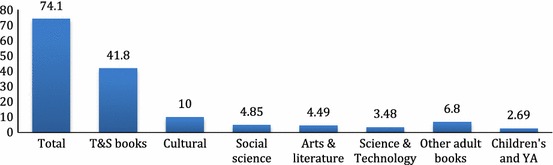
Book sales by Xinhua in 2015 (billion, RMB). *Source*: [1]

Today, in terms of sales, Xinhua is the largest bookseller in China, but whether or not it is the largest book ‘retailer’ is difficult to determine, as over half of Xinhua’s book sales are generated through the non-retail market. In 2015, Xinhua bookstores across China sold a total of ¥74.1 billion worth of books, of which 56 per cent (41.8 billion) were textbooks and school supplementary materials—*jiaofu jiaocai*, or T&S books—which are sold primarily through the educational book distribution channel rather than the retail market. This means that in 2015 Xinhua’s share in the retail book market in China was around ¥32.3 billion (See Fig. 2). Since there are no official data on the sales produced by private booksellers, it is difficult to know what kind of market position this 32.3 billion sales gave to Xinhua. But, a widely agreed estimate is that in today’s retail book market in China, Xinhua tends to account for about 40 per cent, which is surpassed by online retailers by about 5–10 per cent (i.e. online sales account for 45–50 per cent), with private booksellers combined accounting for the remaining 10–15 per cent.

As shown in Fig. 2, Xinhua relies heavily on the sales of T&S books. This is also attested to by the fact that of the total 6.58 billion copies of books sold by Xinhua in 2015, a staggering 67 per cent were T&S books [1]. Though there are no official data on Xinhua’s profit margins, it is commonly believed that T&S books tend to contribute to 60–70 per cent of Xinhua’s overall profit [2–4].

The high proportions of T&S books in Xinhua’s sales and profit margins are a manifestation of the advantageous position the state bookseller occupies in the Chinese bookselling market. T&S books, especially school textbooks, are considered the most profitable type of book in China—both for publishers and for distributors/retailers—and Xinhua has been the sole distributor for school textbooks for decades. Nowadays, some provinces allow private companies to bid for the right to distribute school textbooks, but Xinhua’s financial clout as state-owned companies and long-term monopoly in the field mean that private firms can hardly win such bids. Xinhua, therefore, will continue, at least in the short term, to enjoy and benefit from its privilege in the distribution of textbooks.

As the state-owned bookseller, Xinhua bookstores also enjoy many other privileges. For example, most Xinhua bookstores own their real estate (if not, have the right to rent their premises from their local governments at discounted prices). This means that not only do many Xinhua bookstores operate on a rent-free basis, but they can generate handsome income by letting out part of their real estate. Furthermore, Xinhua bookstores in prefectural-level towns and rural areas have been exempt from VAT for decades, whereas the same policy was not made available to private booksellers until 2013. These privileges have been crucial for the state bookseller to survive in China’s competitive book retail market.

For about 20 years since the mid-1950s, Xinhua had been the only bookseller existing in China. But its dominant position began to change in the late 1970s, when private booksellers, who had previously been forbidden to exist, began to appear in China, and would grow at a rapid rate throughout the following two decades.

### Private Booksellers

The emergence and development of private booksellers in the late 1970s took place against the backdrop of the development of the private economy in China at the time. For much of the three decades since the foundation of the People’s Republic of China in 1949, private businesses were banned in the country. With new leader Deng Xiaoping rising to power and beginning the economic reform in the late 1970s, however, small individually run businesses began to appear and develop rapidly across the country.^2^ By the end of the 1970s, there were over 310,000 individually run businesses [6]. The private economy continued to develop throughout the 1980s and over 14.5 million non-state businesses had been set up by the end of the decade (ibid.: 150–152).

Among the fast-growing body of private businesses was the first generation of private booksellers. Their emergence and development was driven and hastened by the public’s high demand for books and booksellers, in the wake of the end of the Cultural Revolution in 1976. During the Cultural Revolution (1966–1976), the only books available to most ordinary Chinese people were works by Chairman Mao Zedong, as publishers were not allowed to publish many other books and many Xinhua bookstores were closed [7]. As a result, when the Cultural Revolution ended after the demise of Mao in 1976, the public’s demand for books exploded. In this context, private booksellers appeared and flourished across China, first in the form of small book vendors, and then more bricks-and-mortar shops opened. It is estimated that by 1987 there were over 10,000 private booksellers in China, about 1.18 times more than Xinhua’s outlets ([8]: 53).

The retail book market in China in the 1970s and 1980s was a typical seller’s market. Readers would line up outside bookshops overnight to buy books, and “[practically] any book available was welcomed and would succeed” [9]. Bookselling at the time was “a lucrative business that could not fail”, and many were attracted to the business exactly because of this [10]. Most private booksellers in those decades were therefore highly commercially driven and tended to see bookselling as “nothing more than a mere way of making a living”, said a bookseller I interviewed who opened his bookstore in the late 1980s but considered himself different from most of his peers in terms of motivation. Driven by the desire to make money from bookselling, many private booksellers in the 1970s and 1980s ended up selling pirated books and pornography books for high profit (ibid.). Private booksellers, therefore, did not earn themselves too good a reputation in the first 10 years of their development.

The 1990s saw the development of private booksellers both in terms of quantity and in terms of quality. Their numbers increased from just over 10,000 in 1987 to 37,374 in 2000, almost four times as many as Xinhua’s outlets [8, 11]. Some companies had expanded their businesses and a number of regional or national chains appeared. Xishu Bookstore, for example, had over 600 chain or franchise stores across China in its heyday in the late 1990s and early 2000s. Most of these chain booksellers, including Xishu, however, would have closed or downsized by the end of the 2000s.

Another important development in private bookselling in the 1990s was the development of booksellers dedicated to high-quality books. These quality booksellers were a contrast to the mercenary private booksellers in the preceding decades. Since the early 1990s, many of what would have come to be known as ‘academic booksellers’ (*xueshu shudian*) began to appear in China. Examples included All Sages Bookstore in Beijing, opened in 1993 by two Peking University (PKU) graduates, and Sisyphe Bookstore, also founded in 1993 by a PKU graduate, in a small south-western city called Zunyi. It is believed that more than 1500 such ‘academic booksellers’ had opened across China during the 1990s [3]. These booksellers were called ‘academic booksellers’ because they specialized in *xueshu shu*, or ‘academic book’, which in China specifically refers to books in the fields of the social sciences and humanities, especially philosophy, history, political science, sociology, law, and so on.^3^ The scholarly content made ‘academic books’ highly regarded by the public; and for their passion for and dedication to ‘academic books’, ‘academic booksellers’ were esteemed a respectable group of booksellers [12]. Naturally, the rise of ‘academic booksellers’ helped to change the public’s perception of private booksellers and hence reshaped the retail book market and book culture in China.

The 2000s was a turning point for China’s private booksellers. It was in this decade that after more than 20 years of rapid growth, private booksellers began to decline. 2001 saw the first significant drop in the number of private booksellers for over a decade, with the number decreasing from 37,374 in 2000 to 36,448 (see Table 1). The number continued to fall in the following 2 years, resulting in fewer private booksellers in 2003 than in 1996. There are no official data on the numbers of private booksellers after 2003, as the SAPPRFT (the State Administration of Press, Publication, Radio, Film and Television, the highest state authority in charge of the book industry in China) no longer provided such data, but practitioners widely agreed that private booksellers declined significantly throughout the 2000s.

**Table 1:** Numbers of private booksellers, 1994–2003. *Source:* [3, 28]

Year	Total number	Annual change
1994	29,669	n/a
1995	33,415	+3746
1996	35,534	+2119
1997	35,827	+293
1998	35,450	−377
1999	35,282	−168
2000	37,374	+2092
2001	36,448	−926 (2.4%)
2002	36,035	−413 (1.1%)
2003	34,384	−1551 (4.3%)

**Fig. 3:**
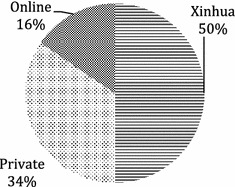
Market share of book retailers in 2014. *Source*: [13–15]

In terms of market share, in 2002 private booksellers still accounted for over half (56.94 per cent) of China’s non-textbook market [3]. This was partly because online retailers did not have substantial development in China until 2003—for reasons I shall explain shortly. But the percentage fell to about one-third in 2014 (See Fig. 3).^4^ Given the rapid growth of online booksellers in recent years—sales increased by 23.5, 33.3 and 31 per cent in 2014, 2015 and 2016 respectively [16]—the market share of private booksellers today should be well below 30 per cent. Data from publishers supported this estimate: for most publishers I interviewed in 2015, the proportion of their book sales by Xinhua, online and private booksellers tended to be 5:3:2 or 4:4:2. With the steady increase in online book sales, it may not be long before the market share of Xinhua, online and private booksellers to become 4:5:1, if not yet.

### Online Bookselling

The history of online bookselling in China began at the turn of the twentieth and twenty-first centuries, marked by the opening of two bookselling websites: Dangdang.com in November 1999 and Joyo.com in early 2000. Yet, neither of the two booksellers had any real impact on the Chinese retail book market until 2003. In that year, due to the outbreak of the SARS (Severe Acute Respiratory Syndrome) virus, in order to minimize the risk of infection by going to crowded places like supermarkets, many Chinese chose to shop online. This accidentally boosted the use of the Internet in Chinese households and led to the rapid growth of online retailers, including online booksellers like Dangdang and Joyo [17, 18]. In April 2003, Dangdang saw 100 per cent increase in traffic and 30 per cent in sales compared to April 2002, whereas Joyo reported to have more sales in the first week of May 2003 than its normal sales in a month [19].

By the end of 2003, Dangdang had ¥100 million sales, making it the largest online book retailer in China. Today, it accounts for around 40 per cent of the online bookselling market in China, approximately the sum of sales by competitors Amazon.cn and JD.com. As the first and largest online book retailer in China, Dangdang resembles Amazon.com in many respects, especially in terms of its struggle to achieve profitability in its early years. Like Amazon, who had run at a loss for 8 years since launching in 1995 [20], Dangdang had operated on a loss-making basis for nearly a decade. The bookseller did not report its first net annual profit of ¥10 million until 2009. In 2010, Dangdang listed on the New York Stock Exchange (NYSE). Yet, right after its listing, the bookseller returned to loss-making: it lost ¥228 million, ¥444 million, and ¥143 million in 2011, 2012, and 2013. The company did not achieve profitability again until 2014, in which year it earned ¥88 million. By this time, however, it is difficult to know how much of Dangdang’s profit was generated from bookselling, as the bookseller had diversified into general retailing since 2004. Just like Amazon.com, Dangdang is now an online retailer selling not only books but also merchandise ranging from household appliances to clothes.

**Fig. 4:**
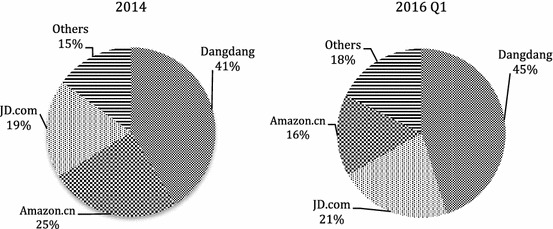
Market share of major online book retailers in 2014 and 2016 Q1. *Source:* [26, 27]

At about the same time as the founders of Dangdang.com were preparing for the launch of their website in 1999, Kingsoft, one of the earliest IT companies in China, was busy turning its loss-making website Joyo.com from an IT service provider into a retail website specializing in books, CDs and videos. Opening in 2000, Joyo was the second online bookseller in China. Unlike Dangdang, Joyo broke even almost immediately after opening and began to generate a profit in 2003. In 2004, Amazon.com acquired Joyo.com for $75 million and turned it into its official Chinese website. In 2007, the company launched its new website Amazon.cn to replace Joyo.com and began to use the name ‘Joyo Amazon’, which it replaced with ‘Amazon China’ in 2011. However, neither Joyo Amazon nor Amazon China was able to replicate in China the success of other members of the Amazon family in other book markets in the world. In 2014, Amazon.cn had market share which was considerably smaller than that of long-term rival Dangdang, and precariously close to that of newcomer JD.com (See Fig. 4). Today, statistics show that Amazon.cn has already been overtaken by JD.com. Like Amazon.com and Dangdang, Amazon.cn is also a general retailer. Yet, its market share in general online retailing is even smaller than its share in bookselling. In 2015, it accounted for only 0.9 per cent of China’s online retail market, negligible compared with Alibaba-run Tmall’s 58 per cent and JD.com’s 22.9 per cent [21]. Given the two companies’ predominance in the general online retail market, it is not surprising to find that they are playing an important—and increasingly so—role in the Chinese online bookselling field.

Founded in 2004 to sell primarily electric products like computers and mobile phones, JD.com is dissimilar from Dangdang and Amazon.cn for the fact that rather than being a bookseller who expanded into general retailing, it is a general retailer who diversified into bookselling. JD.com entered bookselling in 2010, a whole decade after the two trade leaders, but within only 6 years, it had surpassed Amazon.cn and become the second largest online book retailer in China. The rise of JD.com changed the field of online bookselling in many ways, one of which was that its price-cutting-based competition strategy is believed to have normalized the practice in the industry.

Although both Dangdang and Amazon.cn began to discount their books on the first day of opening, neither of them attempted to discount to a loss-making degree just in order to beat one another. The usual discounts the two booksellers gave in the past were in the region of 15–28 per cent, comfortably below the discounts they received from their suppliers (around 40–45 per cent from publishers). However, when JD.com began to sell books in 2010, which was widely believed to be the company’s strategy to use books as loss leaders to attract customers to visit its website and buy other merchandise, it adopted a much more aggressive approach to discounting. In order to compete directly with Dangdang, JD.com not only matched the trade leader’s prices, but it also introduced some new sales methods, such as ¥50/100 off when spending ¥100/200. These methods could mean an additional 50 per cent off on top of the existing discounts and had never been used before by Dangdang or Amazon.cn, as they almost meant outright loss-making by rendering selling prices lower than cost prices.

On 14 December 2010, less than a week after Dangdang’s listing on NYSE, JD.com announced that it would reduce the prices of all its books for them to be 20 per cent cheaper than Dangdang’s prices. Dangdang reacted immediately by adjusting its prices accordingly, so that they were the lowest again. It also for the first time offered the ¥100 off when spending ¥200 deal, and announced that it had raised ¥40 million to fund this ‘sale’. A few hours later, JD.com said its budget for the promotion was ¥80 million. The next day, Amazon.cn joined the battle by also reducing its prices by 20 per cent. The aggressive discounting by the three websites led to a storm of protests by publishers and physical booksellers. One week later, the General Administration of Press and Publication (GAPP, the former SAPPRFT) stepped in and ordered the three companies to stop their sales. However, what the GAPP could not stop was the three leading online retailers’ increasing use of the price-cutting practice in competition. Since 2010, Dangdang, Amazon.cn, and JD.com have been involved in a number of price wars, which led to the normalization of the high discount of 28–35 per cent and the regular special sales (sales that all three online retailers give a number of times throughout the year) in which average discounts can be at least 50 per cent.

A young yet increasingly important player in the Chinese online bookselling business is Tmall Books. Opened in June 2012 as the book department of Tmall.com,^5^ Tmall Books has grown exponentially over the last few years. In 2015, it reported ¥7.5 billion sales, which was three times larger than that of JD.com [22]. Yet, these books were not sold *by* Tmall Books but rather *through* it, as unlike Dangdang.com, Amazon.cn, and JD.com, Tmall Books is not a bookseller but rather a platform for booksellers, publishers, and wholesalers to set up stores and sell books via their Tmall stores. Although the other three retailers also run similar third-party seller markets, Tmall Books has attracted far more sellers than the others—over 2800 in 2015, which, combined, sold more than 400 million copies of books to over 80 million readers (ibid.). Dangdang and Amazon.cn opened their own Tmall stores in 2012 and 2015 respectively. Dangdang’s Tmall store is currently the largest bookstore on Tmall by sales and sold over ¥300 million worth of books in 2015, which amounted to approximately five per cent of Dangdang’s total book sales in the year. Due to its direct competition with Tmall.com in general retailing, JD.com has not opened a store on Tmall Books.

Despite its phenomenal growth, Tmall Books has not presented itself as yet another relentless newcomer whose existence serves only to aggravate the already competitive online bookselling market and give physical booksellers and publishers one more reason to worry. On the contrary, many people—practitioners and commentators—believe that Tmall Books’s rise could actually benefit booksellers and publishers alike. For publishers, they argue that by allowing publishers to open their own online bookstores, Tmall Books could help arrest the price-cutting practice adopted by the big online retailers. The theory is that since publishers could now sell books to customers on their own via the Internet, they can rely less on Dangdang and the like and hence have more power to negotiate with them when demanded for unfair terms. This theory, however, has not been entirely testified on the part of publishers, as all publishers that I interviewed said that sales through Dangdang, JD, and Amazon China were still highly important to them. Despite this, however, they did all state that their organizations either had already or were to set up bookstores on Tmall, as they believed that it was important to do so for it could give them ‘more initiative’ when it comes to selling books online.

For physical booksellers, especially wholesalers, the advent of Tmall Books could prove to be a turning point for their businesses by enabling them to grow again in the face of Dangdang and the like. In a large book wholesale center I visited in Beijing in 2014, over half of the wholesalers were selling books via Tmall. Many told me that they were selling more books through Tmall than in their physical shops. Over half of the top 10 largest bookstores on Tmall Books in 2015 were in fact book wholesale companies that traditionally trade at a book wholesale center.^6^ However, selling books on Tmall may not be as helpful to private retailers as it is to wholesalers. This is because the lower discounts retailers receive—28 to 35 per cent—can hardly allow them to give a discount that is competitive online, which needs to be at least 20 per cent.

### A Future That is Not Necessarily Uncertain

In this article, I have mapped out how the book retailing industry in China has evolved since the late 1970s, with a focus on the development of the three major types of players in the field. It is apparent that in terms of the strong presence of state-owned enterprises, the Chinese book trade differs significantly from the book trades in many other countries in the world, especially the major Western societies like the UK and the US. However, if we look at some newest developments in the industry, we might find that some of these changes resonate with certain trends that are taking place in the West.

For example, in its latest report on the Chinese book market, the German Book Information Centre in Beijing lists “(independent) bookstore fever” as one of the three most significant trends that have occurred in the Chinese book world in recent years (BIZ Peking [23]). “For physical bookstores, 2015 was a year full of hope,” the report acclaims (ibid.: 6).

Sales increased while “bookstore killers”, e-commerce companies, Amazon and Dangdang also wanted to open bookstores. The plans for bookstores by the latter also involved considerable spending (Dangdang plans to open 1000 bookstores in the next 3 years). It is not the first time that we have heard similar ambitious plans. Phoenix-Power, which began as a book wholesaler, has opened a chain of more than 20 bookstores called Belencre (字里行间) in the past 5 years, but this is still a far cry from the 100 in their initial announcement. […] Veteran brand CITIC Books […] too announced in 2015 the setting up of 1000 new bookstores. (ibid.)

From these statistics, a ‘booming’ of bricks-and-mortar booksellers seems to be occurring in China. Such (seemingly) revival of the physical bookstore should not be unfamiliar to observers of the book industry in the West. What I refer to, however, is not—at least not only—the similar business plans at the online bookselling giants—Amazon.com in the US and Dangdang.com in China—to further expand their territory in book retailing by launching more new physical shops. Rather, what I mean here is the increase in the number of high street bookshops opened up by individuals or firms that are not a chain, a phenomenon which has been observed over the past few years on both sides of the Atlantic. In the US, the American Booksellers Association has been celebrating growth in its membership since 2009 [24, 25]. In the UK, according to the Booksellers Association, in 2017 the number of independent bookshops in the UK and Ireland increased, rather than decreased, for the first time in twenty-two years since 1995. It seems that, like their Western counterparts, China’s physical booksellers, after years of declining since the arrival of online retailers in China in the early 2000s, are poised for fighting back. It seems.

Indeed, as BIZ Peking’s report points out, one issue that may hinder this ‘booming’ of the physical bookstore in China is the fact that, many of the newly opened bookshops are in fact operating at a loss. This, without a doubt, adds a less bright note to the long-expected ‘revival’ of the bricks-and-mortar bookseller. The thing is, rather than making a profit—either from selling books or from the sidelines of various types that almost all bookstores run nowadays, which range from coffee shops and non-book merchandising to premium membership schemes—many of China’s bricks-and-mortar booksellers depend for their survival on subsidies from owners, investors’ money, and/or favourable business terms such as free rents. BIZ Peking’s report observes:

Given excellent overall conditions, one cannot help but point out that most of these bookstores have yet to turn a profit. For instance, CITIC Books has always been loss-making but its losses are reportedly narrowing. […] This is despite the fact that the Chinese government has been providing subsidies and VAT exemptions to bookstores since 2014. Elsewhere, many new shopping centers, for the purpose of attracting business tenants, have also given five-year rent-free preferential treatment to bookstores. BIZ Peking [23]

Therefore, what is distinctive about the current wave of bookshop booming in China is that it is driven primarily by some exogenous incentives, in particular government’s tax reduction policy, the investment market’s interest, and large shopping malls’ business strategy. Nevertheless, while these incentives are clearly quite effective, whether or not they are going to be long-term, nobody knows. This hence brings to the fore the question to what extent the model behind the bookshop ‘boom’ is sustainable, which itself raises the question about the nature of this ‘boom’—is it going to lead to a real revival of the bricks-and-mortar bookstore, or is it just a passing fad?

Nobody knows the answers to these questions until the truth reveals itself in the future. Therefore, for any careful observers of the Chinese book retailing market, a shadow of doubt is always lurking about and prevents them from making any definitive predictions about the future of the trade. However, if there is anything that we can learn from the history of the industry, it is likely to be the understanding that, notwithstanding one of the oldest trades there is—having existed for over 2000 years in China [7]—the book trade is more resilient to new changes than it may first appear to be. That is, it is very doubtful that the (physical) bookstore will disappear from our society altogether. In the meantime, book retailing, a special kind of business activity that entails as much exchange of human knowledge, experiences, ideas, and even emotions as the transaction of material goods and finances, is too deeply grounded in interpersonal communication and interaction to be wholly fulfilled by computers and algorithms. Dangdang’s ambition to open one thousand physical shops, for example, is revealing—perhaps more revealing than the recent spate of bookshops on the Chinese high street driven by the various rather contingent factors—about the vitality of bricks-and-mortar book retailing. Therefore, while the future of the book retailing industry in China may be unpredictable, it is not necessarily uncertain. Rather, what may be anticipated, based on what is learnt from the past, could be that after years of adjusting and reorientation, the moment for the book retailing industry to redress its balance between bricks-and-mortar bookselling and online retailing, and for the power relation between physical booksellers and online retailers to rebalance, has finally arrived in China.

## Footnotes

1 I focus in this article on the general interest book sector, that is, books published for and sold through the mass consumer market (children’s books included). My account presented in this article may not necessarily be applicable to the more special sectors, such as the educational book market.

2 An important reason for the government to allow private businesses to exist was that after the Cultural Revolution ended in 1976, millions of young Chinese who had been sent to the countryside during the Revolution as part of a socialist campaign began to return to their home cities, but many soon found themselves unemployed. Many such ‘returned youth’ thus turned to self-employment by running small businesses like dressmaking and repairing. The central and local governments tolerated these businesses because they helped alleviate the massive unemployment rampant in many Chinese cities at the time. For more about the role of the ‘returned youth’ in the development of the private economy in China during the late 1970s, see Coase and Wang, *How China Became Capitalist* [5].

3 Because the boundary between trade and academic publishing was (and is) not clear-cut in China, an ‘academic book’ can refer to a scholarly monograph written by and for academics or a trade non-fiction written for a more general readership but with scholarly content.

4 These figures were calculated based on the following data: the total book sales in China in 2014 were estimated to be 136.8 billion yuan [13], of which Xinhua accounted for 68.5 billion [14] and online retailers 21 billion [15]. Taken together, this left about 47.3 billion worth of books sold by private booksellers, amounting to around 34.5 per cent of the market.

5 Tmall.com is an e-commerce website run by Alibaba Group. Thanks to parent company Alibaba’s large customer base—over 423 million active users as of March 2016 [22]—Tmall.com is the largest online retailer/retail website in China, which has clearly benefited the rapid growth of Tmall Books.

6 In China, a book wholesale center is a trading place—normally a substantial building similar to a shopping mall—in which book wholesale companies can rent individual shops to store their stock and to trade face-to-face with customers—mainly retailers, though the public are also served.
